# Comparison of gastrointestinal adverse events between fast release tablets and regular acetylsalicylic acid (aspirin) galenics after short-term use: a meta-analysis of randomized clinical trials

**DOI:** 10.1007/s10787-023-01264-3

**Published:** 2023-08-21

**Authors:** Angel Lanas, Oliver Werz, Engy Mikhail

**Affiliations:** 1 University of Zaragoza, Aragón Health Research Institute (IIS Aragón), CIBERehd, Saragossa, Spain; 2 Department of Pharmaceutical/Medicinal Chemistry, Institute of Pharmacy, Friedrich Schiller University Jena, Jena, Germany; 3 Bayer Consumer Health, Whippany, NJ USA

**Keywords:** Fast releases aspirin, Regular formulatons of aspirin, Short-term treatment, Gastrointestional adverse events

## Abstract

This study aimed at determining whether there is a difference in the safety profile between fast release (FR) aspirin tablets and regular galenic formulations of aspirin. This study was based on a clinical study database pool (Bayer HealthCare) including 84 clinical studies and 16,095 human subjects. The meta-analysis included 72 studies applying a single dose of aspirin of at most 1000 mg and was, therefore, based on individual data from 9288 subjects. Of these, 6029 subjects took aspirin and 3259 subjects took placebo. Endpoints were adverse events (AEs) of any kind and, especially of gastrointestinal (GI) nature. Event incidence and odds ratios (OR) based on Mantel–Haenszel risk estimates were calcuated. Subjects on aspirin FR had a significantly decreased OR of 0.65 [0.48, 0.90] [95% confidence interval] for all AEs and of 0.39 [0.20, 0.79] for drug-related all AEs versus placebo. The risk of all GI AEs tended to be reduced for subjects on aspirin FR (0.65 [0.41; 1.03]), but not for drug-related GI AEs. Subject on aspirin mono and aspirin mono (plain only, w/o FR) showed an increased risk of drug-related all AEs compared to placebo (1.34 [1.11; 1.62] and 1.43 [1.13; 1.80]). However, subjects on aspirin FR and those on regular aspirin had almost the same risk of all determined AEs. In conclusion, aspirin FR tablets showed a comparable GI tolerability to regular galenic formulations of aspirin after short-term treatment. Major GI complication did not occur after intake of any galenic formulation of aspirin.

## Introduction

Aspirin is one of the most commonly used over-the-counter (OTC) non-steroidal anti-inflammatory drug (NSAID) worldwide which is associated with the generic name acetylsaliylic acid (ASA) (Forder et al. 2016; Gurbel et al. 2019). ASA exhibits anti-inflammtory, analgesic, anti-pyretic, and antithrombotic properties. The main mode of action is based on the non-selective inhibition of cyclooxygenase (COX)-1 and COX-2 enzymes leading to a significant reduction of prostaglandin and thromboxane synthesis (Bianconi et al. 2020). The indications for short-term OTC usage of aspirin are mild-to-moderate painful symptoms such as headache, dental pain, sore throat as well as fever or symptoms associated with the common cold. Therapeutic dosage for these indications of short-term aspirin use is generally 325–1000 mg repeated at 4–6-h intervals up to 3 g per day (Bayer Consumer Health 2022). Aspirin-based products are available in different galenic formulations such as plain tablets, effervescent tablets, granules or fast release (FR) tablets. These galenic formulations affect both the pharmacokinetic and pharmacodynamic actions of aspirin for the given indication and, therefore, also influence efficacy and safety. Aspirin FR tablet is a recently devolped fast disintegrating and dissolving galenic formulation which is characterized by two improvements. First, the tablet core contains sodium carbonate which acts as a superdisintegrant in the acidic milieu of the stomach and increases disintegration of the tablet. Second, the active ingredient is micronized which contributes to a faster dissolution. These modifications lead to an earlier onset of drug plasma concentrations and action compared to previous formulations (Voelker and Hammer 2012; Cooper and Voelker 2012; Voelker et al. 2016; Stevens et al. 2019).

NSAIDs cause adverse events (AEs) in several organs, but most frequently this occurs in the upper and lower gastrointestinal (GI) tracts (García–Rayado et al. 2018). So far, most studies investigated the GI safety profile of long-term use of low-dose aspirin for prevention of cardiovascular events and reported an increased risk of major bleeding (García Rodríguez et al. 2016; Whitlock et al. 2016). However, data on the GI side effects of short-term use of aspirin are rare. A former meta-analysis based on a clinical study database pool including a total of 67 trials investigated the safety profile of a short-term use of regular galenic formulations of aspirin at the recommended doses for various OTC ASA indications. This meta-analysis reported a slight increase in the risk of mild to moderate dyspepsia and abdominal pain with aspirin compared to placebo, but major GI complications were not observed (Lanas et al. 2011). However, this meta-analysis only investigated regular galenic formulations of aspirin, but not aspirin FR tablets. It was assumed that the galenic formulation of aspirin FR should have fewer side effects than regular galenic formulations of aspirin especially due to a fast passage time in the stomach and a different pharmacokinetic profile, but this is not known and poorly evidenced.

Therefore, the present study is an update of the above-mentioned meta-analysis including the same study database pool of 67 clinical studies plus 17 studies which for the most part investigated the safety profile of aspirin FR tablets. To fill a scientific gap for aspirin on concrete product level, the main aim of this study was to determine whether there is a difference in the safety profile between aspirin FR tablets and regular galenic formulations of aspirin.

## Patients and methods

### Setting

A former meta-analysis based on a clinical study database (Bayer HealthCare) generated by March 31, 2008 included a total of 67 clinical studies, where adequate data documentation in terms of AE reporting was available (Lanas et al. 2011). For the current update, 17 additional studies conducted after March 31, 2008 were added to the clinical study database pool and analyzed together with the former trials. In ten of these studies, aspirin FR tablet was investigated and thus, data from 796 subjects were available to assess the frequency of side effects of the latest formulation of aspirin. In total, this database pool included data of 16,095 human subjects from 84 studies. In contrast to the former meta-analysis, studies with ASA doses ≤ 325 mg were included. The most relevant inclusion and exclusion criteria in these studies have already been described before (Lanas et al. 2011).

### Endpoints

The subjects were asked to report any AE and investigators were instructed to give a clinical diagnosis of the AEs. An AE was considered as treatment-emergent adverse event (TEAE) if it had occurred after treatment on the day of treatment or up to 7 days thereafter. The TEAEs of all studies (former studies and additional studies) were coded using the current Medical Dictionary for Regulatory Activities (MedDRA), Version 23.0 either according to predefined, standardized MedDRA queries or according to selected MedDRA preferred terms, high-level terms, high-level group terms, or system organ class. Bayer HealthCare assigned the appropriate MedDRA term to each AE. The intensity of the AEs was defined as mild, moderate, or severe. The following events of interest were defined based on the overall number of AEs and the known GI side effect profile:

- **All AEs:** all AEs in any system organ class (SOC) and any preferred term (PT),
- **All GI AEs:** all AEs with SOC “Gastrointestinal disorders” and any PT,
- **Dyspepsia:** all AEs with PTs “Dyspepsia,” “Epigastric discomfort,” and “Eructation,”
- **Minor GI AEs:** all AEs with PTs “Heartburn,” “Nausea,” “Vomiting,” and “Abdominal pain,”
- **GI bleeding:** all AEs with PT “Haematemesis,”, “Haematochezia,” and “Melaena.”

In an additional analysis, the study investigators identified AEs that were related to the study drug. Drug-related AEs were defined as those AEs for which the relationship to the study drug was reported by the study investigator as at least possible, i.e., as yes, possibly, probably or definitely. Events that were reported to be unlikely or not related were not considered as drug-related AEs. Individual events that had no relationship reported were considered as drug-related AEs. However, if the relationship had not been collected for the entire study, the study was excluded from the drug-related AE analysis.

### Treatments considered for the analysis

Various subject populations were included in the integrated database, both healthy subjects and patients. The following treatment groups were defined:

- 1.aspirin mono which included all available galenic formulations of aspirin alone or in combination with vitamin C, caffeine, calcium, etc.
- 2. aspirin + pseudoephedrine (PSE) oral granules which is used for the treatment of swelling of the nasal mucosa and paranasal sinuses during common cold in combination with pain and fever (Bayer Vital GmbH 2021),
- 3. aspirin FR tablets,
- 4. aspirin mono without (w/o) FR tablets and only plain tablets, and
- 5. placebo.

This study reported on comparisons between the following treatment groups “aspirin mono versus (vs.) placebo,” “aspirin + PSE vs. placebo,” “aspirin mono (plain, w/o FR) vs. placebo,” “aspirin FR vs. placebo,” “aspirin mono (plain, w/o FR) vs. aspirin FR.”

### Data extraction and management

Data management and statistical evaluation were performed using the Statistical Analysis System (SAS^®^) software package version 9.3 (SAS Institute Inc., Cary, NC, USA). The database structure was based on agreement between Bayer HealthCare and M.A.R.C.O. GmbH & Co. KG, Duesseldorf, Germany, an independent institute for clinical research and statistics. Data for the new studies were provided by Bayer HealthCare in one of the following formats: SAS^®^ datasets for 16 studies and text format for one study. Data for the former studies were provided in formats as described elsewhere (Lanas et al. 2011). In a next step, the SAS^®^ data sets were transformed into the target database structure using SAS^®^ modification programs. Information concerning study title, design, blinding, randomization, dosing, and so forth was partially contained in the data files. Otherwise, it was derived from the study reports and integrated into the target database. At each data management step, appropriate quality control checks were in place.

### Statistics

The scope of the analysis, statistical methods, and content of tables and graphs were laid down in a statistical analysis plan (SAP) before the start of the analysis.

The calculation of the incidence rates and the analyses of the ORs were based on the meta-analysis and were restricted to single-dose studies applying an aspirin dose of at most 1000 mg since these criteria apply to all studies. For the parallel group studies, all data were included; for the cross-over studies, only the first period data were included. The incidence rates and OR were calculated for all AEs and separately for GI AEs as defined before. The treatment comparisons were performed as described in the `treatments considered for the analysis´ section. The same analyses were performed restricted to drug-related AEs.

Incidence rates were calculated as number of subjects who reported at least one event in the numerator and the number of all subjects under observation in the denominator. The OR estimator was based on the Mantel–Haenszel risk estimator, as this is robust even in “sparse data” stratifications, i.e., where few cases of AEs occur. OR analyses studies with zero events were combined and one event was added in each group to allow for OR calculation. Heterogeneity was tested. The modified Breslow/Day statistic was applied with regard to the OR (Breslow and Day 1980; Tarone 1985). A *p* value of ≤ 0.10 was considered as a sign of heterogeneity. In this case, an attempt to identify responsible studies was made and a removal of the studies from the analysis set was considered. A continuity correction considering the treatment group sizes was used (Sweeting et al. 2004). This particularly means that in case of equally sized treatment groups 0.05 was added. For cases, where no events were observed in both treatment groups, the OR was undefined. However, in an attempt to include studies where no events were reported in both treatment groups (and no OR could be calculated) in the meta-analysis, such studies were combined by adding the total numbers of subjects by treatment group and by assuming an equal number of one event in each treatment group.

### Descriptive statistics

We determined the incidence rates of subjects with at least one AE separated by treatment and dose group (1) 0–500 mg or (2) 501–1000 mg aspirin mono, aspirin + PSE, aspirin mono [plain only, w/o FR], aspirin FR or placebo). We differentiated between drug-unrelated and drug-related all AEs, all GI AEs, dyspepsia, and minor GI AEs. In addition, we performed this analysis restricted to subjects suffering from sore throat, dental pain and in healthy volunteers, respectively.

## Results

### Clinical study database pool and demographics

**Fig. 1:**
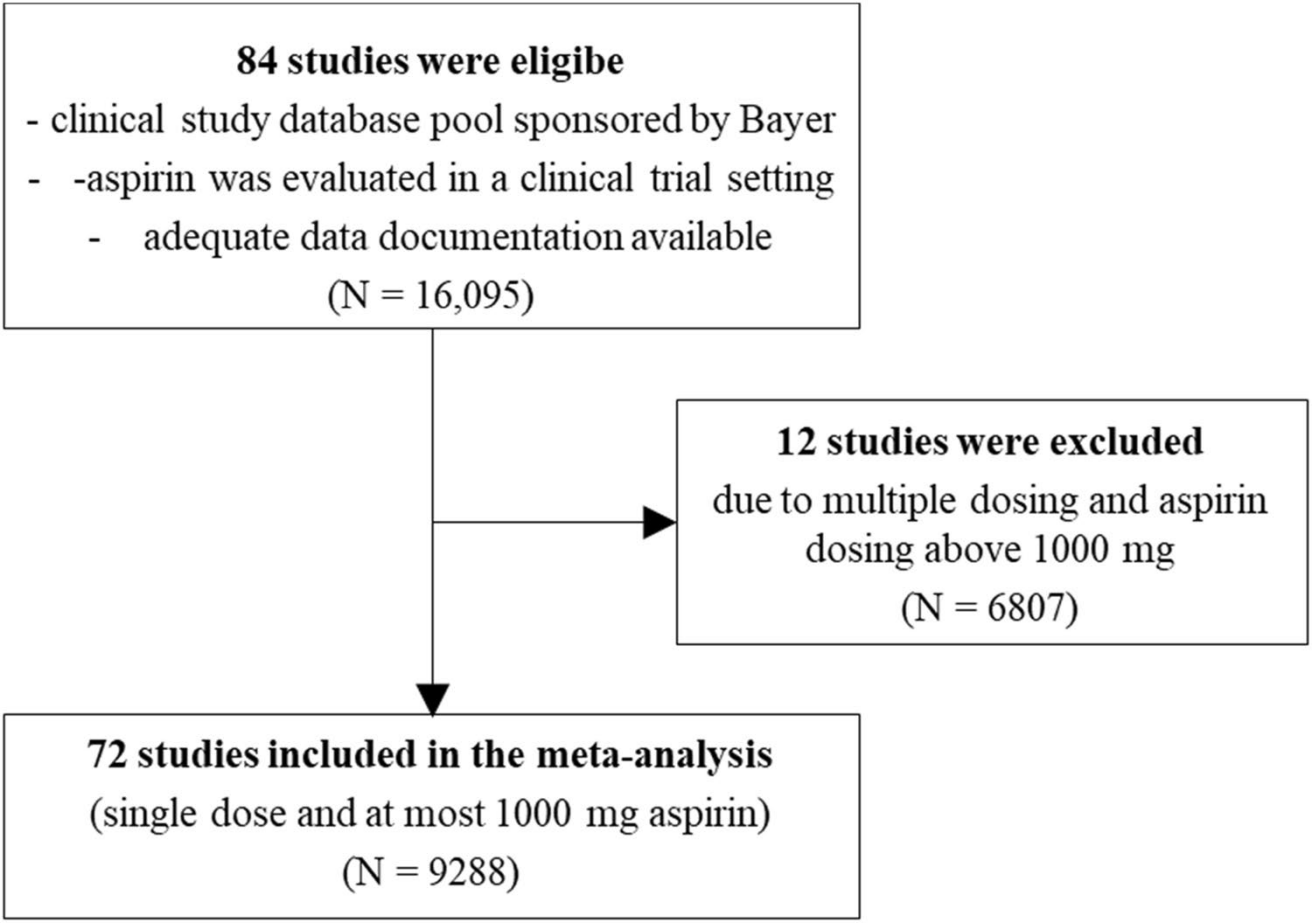
A flow diagram showing the identification of eligible studies. *N* number of subjects

This investigation was based on a clinical study database pool consisting of 84 studies including 16,095 subjects (Fig. 1). Apart from 18 subjects with diabetes, the population consisted of subjects who took aspirin for treatment of pain (*N* = 7174) or common cold (*N* = 6752). There were 2151 subjects of Phase 1 studies who were healthy volunteers (data not shown). The distribution of healthy volunteers was slightly higher in the aspirin mono group (20.36%) compared to the aspirin + PSE (13.06%), aspirin mono (plain only, w/o FR) (14.11%), and aspirin FR (14.45%) groups. The placebo group included slightly less healthy volunteers (8.92%) (Online Resource 1). Twelve studies were excluded due to multiple dosing and aspirin dosing above 1000 mg (6807 subjects). Therefore, the meta-analysis included 72 studies applying a single dose of aspirin of at most 1000 mg and was, therefore, based on individual data from 9288 subjects (Fig. 1). An overview of involved studies separated by parallel and cross-over design is shown in Online Resource 2. Of the 72 studies involved in the meta-analysis, 30 studies (8261 subjects) were based on a parallel design and 42 studies (1027 sujects) on a cross-over design. 5202 subjects took aspirin mono and 827 subjects aspirin + PSE. Most subjects took aspirin as plain (3091 subjects) or in liquid form (1794 subjects). 796 subjects took aspirin FR. Placebo was administered to 3259 subjects.

Table 1 provides an overview of the demographic characteristics of the subjects included in the meta-analysis. In all treatment groups, slightly more women were included than men. The majority of subjects were Caucasian and few subjects were Black, Hispanic, or Asian. Subjects of other ethnic origins were rare. Mean age and BMI were comparable between the aspirin mono and the placebo group. Subjects in the aspirin + PSE group were younger and had a slightly lower mean BMI than those in the other treatment groups.

**Table 1:** Demographic characteristics of subjects included in the meta-analysis

	Aspirin mono	Aspirin + PSE	Placebo	Total
Gender [*N* (%)]				
Total	5202 (100.0%)	827 (100.0%)	3259 (100.0%)	9288 (100.0%)
Male	2152 (41.4%)	410 (49.6%)	1129 (34.6%)	3691 (39.7%)
Female	3050 (58.6%)	417 (50.4%)	2130 (65.4%)	5597 (60.3%)
Race [*N* (%)]				
Total	5202 (100.0%)	827 (100.0%)	3259 (100.0%)	9288 (100.0%)
Missing	835 (16.1%)	2 (0.2%)	294 (9.0%)	1131 (12.2%)
Caucasian	3832 (73.7%)	738 (89.2%)	2659 (81.6%)	7229 (77.8%)
Black	259 (5.0%)	47 (5.7%)	152 (4.7%)	458 (4.9%)
Asian	76 (1.5%)	6 (0.7%)	31 (1.0%)	113 (1.2%)
American Indian	1 (0.0%)	1 (0.1%)	1 (0.0%)	3 (0.0%)
Hispanic	122 (2.3%)	7 (0.8%)	77 (2.4%)	206 (2.2%)
Other	77 (1.5%)	26 (3.1%)	45 (1.4%)	148 (1.6%)
Age [year]				
* N*	5152	827	3209	9188
Mean	32.1	21.4	31.4	30.9
SD	12.5	5.6	12.7	12.5
Range	15–75	18–54	15–72	15–75
Weight [kg]				
* N*	4136	827	2900	7863
Mean	73.0	70.2	71.2	72.0
SD	15.4	14.2	15.2	15.2
Range	35–158.8	41–167.0	40–157.9	35–167.0
BMI [kg/m^2^]				
* N*	4136	827	2900	7863
Mean	25.2	23.3	24.7	24.8
SD	4.8	3.5	4.7	4.7
Range	14.5–60.6	16.6–47.3	12.5–56.2	12.5–60.6

*BMI* body mass index, *N* number of subjects, *PSE* pseudoephedrine, *SD* standard deviation

### Comparison of the risk of adverse events between regular aspirin and aspirin FR

**Fig. 2:**
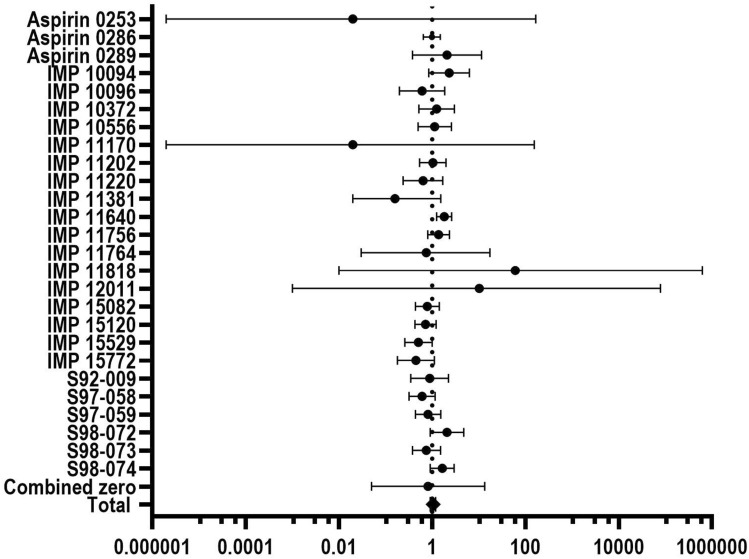
Forest plot summarizing individual study estimates and pooled estimates for the comparison “aspirin mono vs. placebo” for all adverse events. The diamond represents the overall (pooled) association estimate. Bars indicate 95% confidence intervals. Dashed vertical line indicates the pooled random effects estimate

**Fig. 3:**
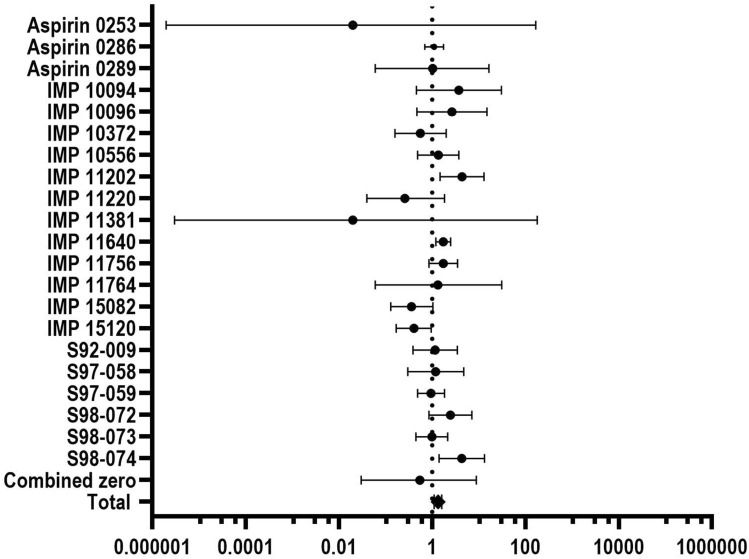
Forest plot summarizing individual study estimates and pooled estimates for the comparison “aspirin mono vs. placebo” for drug-related all adverse events. The diamond represents the overall (pooled) association estimate. Bars indicate 95% confidence intervals. Dashed vertical line indicates the pooled random effects estimate

Tables 2 and 3 present (drug-related) AEs for regular formulations of aspirin and aspirin FR compared to placebo and to each other. Examples for a list of individual study OR and pooled OR for (drug-related) all AEs are provided in Online Resources 3 and 4. Forest plots summarizing individual study estimates and pooled estimates for (drug-related) all AEs are exemplarily shown in Figs. 2 and 3.

**Table 2:** Incidence rates and odds ratios for all (gastrointestinal) adverse events

Event of interest	Comparison	Incidence (%) (response/total)			Odds ratio with 95% CI
		Aspirin group	Control group		
All AEs	Aspirin mono vs. placebo	14.1 (611/4346)		14.4 (404/2802)	1.05 [0.91, 1.20]
	Aspirin + PSE vs. placebo	11.3 (81/716)		10.2 (37/363)	1.23 [0.82, 1.86]
	Aspirin mono (plain only, w/o FR) vs. placebo	13.5 (377/2794)/		13.6 (242/1773)	1.11 [0.93, 1.33]
	Aspirin FR vs. placebo	14.4 (98/681)		20.4 (70/343)	**0.65 [0.48, 0.90]**
	Aspirin mono (plain only, w/o FR) vs. aspirin FR	14.7 (62/421)		15.4 (68/441)	0.93 [0.64, 1.36]
All GI AEs	Aspirin mono vs. placebo	5.5 (238/4346)		6.1 (171/2802)	1.00 [0.81, 1.23]
	Aspirin + PSE vs. placebo	3.5 (25/716)		3.9 (14/363)	1.04 [0.53, 2.05]
	Aspirin mono (plain only, w/o FR) vs. placebo	3.9 (108/2794)		4.1 (73/1773)	0.97 [0.72, 1.30]
	Aspirin FR vs. placebo	5.9 (40/681)		8.7 (30/343)	0.65 [0.41, 1.03]
	Aspirin mono (plain only, w/o FR) vs. aspirin FR	4.8 (20/421)		5.4 (24/441)	0.87 [0.47, 1.61]
Dyspepsia	Aspirin mono vs. placebo	1.5 (64/4346)		1.7 (49/2802)	1.24 [0.83, 1.85]
	Aspirin + PSE vs. placebo	0.3 (2/716)		0.8 (3/363)	0.61 [0.07, 5.37]
	aspirin mono (plain only, w/o FR) vs. placebo	0.8 (22/2794)		0.5 (9/1773)	1.72 [0.80, 3.71]
	Aspirin FR vs. placebo	0.3 (2/681)		0.6 (2/343)	0.50 [0.08, 3.02]
	Aspirin mono (plain only, w/o FR) vs. aspirin FR	0.2 (1/421)		0.5 (2/441)	0.54 [0.05, 5.76]
Minor GI AEs	Aspirin mono vs. placebo	2.7 (118/4346)		3.2 (89/2802)	0.81 [0.61, 1.07]
	aspirin + PSE vs. placebo	1.4 (10/716)		1.9 (7/363)	0.79 [0.30, 2.11]
	Aspirin mono (plain only, w/o FR) vs. placebo	2.1 (59/2794)		2.7 (48/1773)	0.77 [0.53, 1.13]
	Aspirin FR vs. placebo	4.7 (32/681/)		6.7 (23/343)	0.68 [0.41, 1.15]
	Aspirin mono (plain only, w/o FR) vs. aspirin FR	3.8 (16/421)		4.5 (20/441)	0.83 [0.42, 1.63]
GI bleeding	Aspirin mono vs. placebo	0.0 (1/4346)		0.1 (3/2802)	0.23 [0.03, 1.70]
	Aspirin + PSE vs. placebo	0.1 (1/716)		0.6 (2/363)	0.26 [0.03, 2.23]
	Aspirin mono (plain only, w/o FR) vs. placebo	0.0 (1/2794)		0.1 (2/1773)	0.30 [0.04, 2.39]
	Aspirin FR vs. placebo	0.1 (1/681)		0.3 (1/343)	0.50 [0.04, 6.75]
	Aspirin mono (plain only, w/o FR) vs. aspirin FR	0.2 (1/421)		0.2 (1/441)	1.05 [0.06, 17.08]

Only studies applying an aspirin dose of at most 1000 mg (single dose) are included

Significant associations were highlighted in bold

*AE* adverse event, *CI* confidence interval, *FR* fast release, *GI* gastrointestinal, *PSE* pseudoephedrine, *PT* preferred term, *SOC* system organ class

**Table 3:** Incidence rates and odds ratios for all drug-related (gastrointestinal) adverse events

Event of interest	Comparison	Incidence (%) (response/total)		Odds ratio with 95% CI
		Aspirin group	Control group	
All AEs	Aspirin mono vs. placebo^a^	7.8 (337/4346)	7.7 (216/2802)	**1.34 [1.11, 1.62**]
	Aspirin + PSE vs. placebo	5.7 (41/716)	5.8 (21/363)	1.12[0.65, 1.94]
	Aspirin mono (plain only, w/o FR) vs. placebo	8.4 (235/2794)	7.7 (137/1773)	**1.43 [1.13, 1.80]**
	Aspirin FR vs. placebo^b^	1.9 (13/681)	4.7 (16/343)	**0.39 [0.20, 0.79]**
	Aspirin mono (plain only, w/o FR) vs. aspirin FR^b^	3.0 (12/403)	3.0(12/406)	1.01 [0.45, 2.28]
All GI AEs	Aspirin mono vs. placebo^a^	3.5 (150/4346)	3.9 (109/2802)	1.14 [0.88, 1.48]
	Aspirin + PSE vs. placebo	2.1 (15/716)	3.0 (11/363)	0.83 [0.37, 1.87]
	Aspirin mono (plain only, w/o FR) vs. placebo	2.6 (72/2794)	2.4 (43/1773)	1.14 [0.78, 1.67]
	Aspirin FR vs. placebo^b^	1.0 (7/681)	2.3 (8/343)	0.43 [0.17, 1.13]
	Aspirin mono (plain only, w/o FR) vs. aspirin FR^b^	2.0 (8/403)	1.5 (6/406)	1.35 [0.46, 3.92]
Dyspepsia	Aspirin mono vs. placebo^a^	1.3 (58/4346)	1.6 (45/2802)	1.24 [0.82, 1.88]
	Aspirin + PSE vs. placebo	0.1 (1/716)	0.8 (3/363)	0.32 [0.02, 4.91]
	Aspirin mono (plain only, w/o FR) vs. placebo	0.8 (22/2794)	0.5 (8/1773)	1.98 [0.88, 4.46]
	Aspirin FR vs. placebo^b^	0.1 (1/681)	0.3 (1/343)	0.50 [0.04, 6.75]^c^
	Aspirin mono (plain only, w/o FR) vs. aspirin FR^b^	0.2 (1/403)	0.2 (1/406)	1.01 [0.06, 16.20]^c^
Minor GI AEs	Aspirin mono vs. placebo^a^	1.4 (62/4346)	1.6 (45/2802)	0.93 [0.63, 1.38]
	Aspirin + PSE vs. placebo	0.7 (5/716)	1.7 (6/363)	0.47 [0.14, 1.56]
	Aspirin mono (plain only, w/o FR) vs. placebo	1.4 (38/2794)	1.5 (26/1773)	0.93 [0.57, 1.51]
	Aspirin FR vs. placebo^b^	1.0 (7/681)	2.3 (8/343)	0.43 [0.17, 1.13]
	Aspirin mono (plain only, w/o FR) vs. aspirin FR^b^	2.0 (8/403)	1.5 (6/406)	1.35 [0.46, 3.92]
GI bleeding	Aspirin mono vs. placebo^a^	0.0 (1/4346)	0.1 (3/2802)	0.23 [0.03, 1.70]
	Aspirin + PSE vs. placebo	–	–	Not estimated
	Aspirin mono (plain only, w/o FR) vs. placebo	–	–	Not estimated
	Aspirin FR vs. placebo^b^	–	–	Not estimated
	Aspirin mono (plain only, w/o FR) vs. aspirin FR^b^	–	–	Not estimated

Significant associations were highlighted in bold

*CI* confidence interval, *GI* gastrointestinal, *N* number of subjects

^a^Only studies applying an aspirin dose of at most 1000 mg (single dose) are included

^b^Only double-blind studies applying an aspirin dose of at most 1000 mg (single dose) are included

^c^Based on zero event studies only

For subjects on aspirin FR, we found that they had a significantly decreased risk of 35% of all AEs compared to those on placebo. The same applied for drug-related all AEs: subjects on aspirin FR had a significantly reduced risk of 61% compared to the placebo group. For all GI AEs, there was a trend of a decreased risk of 35% for subjects on aspirin FR compared to those on placebo, b this result was not significant, as the confidence interval included one (0.65 [0.41; 1.03]). There was no difference in the risk of drug-related all GI AEs and (drug-related) minor GI AEs between the aspirin FR and placebo groups. For (drug-related) dyspepsia, an appropriate interpretation of the ORs was impossible due to the low numbers of cases.

Subjects on aspirin mono, aspirin + PSE or aspirin mono (plain only, w/o FR) had almost the same risk of all AEs, all GI AEs, dyspepsia, and minor GI AEs compared to the placebo group. For drug-related all AEs, subjects on aspirin mono and subjects on aspirin mono (plain only, w/o FR) had a signicantly higher risk of 34% and 43%, respectively, than those on placebo. Subjects on aspirin + PSE had the same risk of drug-related all AEs compared to those on placebo. For drug-related all GI AEs, dyspepsia, and minor GI AEs, subjects on aspirin mono, aspirin + PSE or aspirin mono (plain only, w/o FR) showed almost the same risk as those on placebo.

Subjects on aspirin mono (plain only, w/o FR) had almost the same risk of all AEs, all GI AEs, and minor all GI AEs as those on aspirin FR. For dyspepsia, an appropriate interpretation of the ORs was impossible due to the low numbers of cases. The same applied for drug-related all AEs, all GI AEs, and minor GI AEs, and dyspepsia.

For the risk of GI bleeding, an appropriate interpretation of the ORs was impossible due to the low numbers of cases (Table 2). The same applied for drug-related GI bleeding (Table 3).

Overall, the heterogeneity test showed that the reported ORs were comparable across the studies in this meta-analysis (homogeneity). For the comparison “aspirin mono vs. placebo” for all AEs, larger deviations from unity occurred only twice in each direction and were due to very small sized studies. Since the majority of the studies showed ORs close to unity, the overall result of the meta-analysis can be considered as valid (Fig. 2).

### Impact of two doses of regular aspirin and aspirin FR on the incidence of adverse events in all subjects

In all aspirin treatment groups, the percentage of subjects with at least one AE was comparable in both dose groups “500 mg aspirin or less” and “501–1000 mg aspirin”. In the aspirin mono and aspirin mono (plain only, w/o FR), there might be a slightly higher percentage of subjects with drug-unrelated or -related GI based AEs in the higher than in the lower dose group.

The incidence rates of subjects with at least one drug-unrelated all GI AEs and minor GI AEs were slightly higher in subjects on aspirin FR than in those on regular aspirin or placebo. However, for drug-related all GI AEs and minor GI AEs, subjects on aspirin FR had the lowest incidence rates compared to the other treatments. Subjects on aspirin mono, aspirin mono (plain only, w/o FR) or placebo showed almost no differences in the occurrence of drug-unrelated and—related all GI AEs and minor GI AEs. Drug-unrelated dyspepsia hardly occurred and drug-related dyspepsia did not occur in the aspirin + PSE and the aspirin FR groups, but there were same drug-unrelated and –related dyspepsia cases in both aspirin mono groups (Table 4).

**Table 4:** Number of subjects (%) with at least one adverse event separated by treatment and dose group—all indications and healthy volunteers

	Aspirin mono			Aspirin + PSE			Aspirin mono, plain only, w/o FR			Aspirin FR			Placebo
	0–500 mg	501–1000 mg	Total	0–500 mg	501–1000 mg	Total	0–500 mg	501–1000 mg	Total	0–500 mg	501–1000 mg	Total	Total
Number of subjects treated	1324	3878	5202	467	360	827	1027	2064	3091	74	722	796	3259
Number of subjects with at least 1 AE	179 (13.52)	490 (12.64)	669 (12.86)	55 (11.78)	41 (11.39)	96 (11.61)	151 (14.70)	241 (11.68)	392 (12.68)	11 (14.86)	99 (13.71)	110 (13.82)	443 (13.59)
Drug-unrelated													
All AEs	56 (4.23)	285 (7.35)	341 (6.56)	33 (7.07)	20 (5.56)	53 (6.41)	38 (3.70)	129 (6.25)	167 (5.40)	9 (12.16)	89 (12.33)	98 (12.31)	227 (6.97)
All GI AEs	8 (0.60)	96 (2.48)	104 (2.00)	6 (1.28)	5 (1.39)	11 (1.33)	6 (0.58)	36 (1.74)	42 (1.36)	2 (2.70)	34 (4.71)	36 (4.52)	67 (2.06)
Dyspepsia	0 (0.00)	11 (0.28)	11 (0.21)	1 (0.21)	0 (0.00)	1 (0.12)	0 (0.00)	1 (0.05)	1 (0.03)	0 (0.00)	1 (0.14)	1 (0.13)	4 (0.12)
Minor GI AEs	3 (0.23)	58 (1.50)	61 (1.17)	2 (0.43)	3 (0.83)	5 (0.60)	2 (0.19)	22 (1.07)	24 (0.78)	1 (1.35)	26 (3.60)	27 (3.39)	46 (1.41)
Drug-related													
All AEs	125 (9.44)	236 (6.09)	361 (6.94)	24 (5.14)	21 (5.83)	45 (5.44)	115 (11.20)	126 (6.10)	241 (7.80)	2 (2.70)	12 (1.66)	14 (1.76)	239 (7.33)
All GI AEs	16 (1.21)	140 (3.61)	156 (3.00)	11 (2.36)	5 (1.39)	16 (1.93)	14 (1.36)	60 (2.91)	74 (2.39)	1 (1.35)	6 (0.83)	7 (0.88)	116 (3.56)
Dyspepsia	5 (0.38)	52 (1.34)	57 (1.10)	0 (0.00)	0 (0.00)	0 (0.00)	5 (0.49)	16 (0.78)	21 (0.68)	0 (0.00)	0 (0.00)	0 (0.00)	44 (1.35)
Minor GI AEs	6 (0.45)	58 (1.50)	64 (1.23)	2 (0.43)	3 (0.83)	5 (0.60)	6 (0.58)	32 (1.55)	38 (1.23)	0 (0.00)	6 (0.83)	6 (0.75)	49 (1.50)

### Effect of two doses of regular aspirin and aspirin FR on the incidence of adverse events in subjects with different kinds of pain and healthy volunteers

In the pain model including subjects suffering only from sore throat, we did not find any differences in the impact of the 500 mg or less and the 501–1000 mg dose of regular aspirin on the occurrence of drug-unrelated and related all AEs or GI based AEs. Subjects on aspirin mono, aspirin mono (plain only, w/o FR) and placebo had comparable incidence rates for drug-unrelated GI disorders, but subjects on aspirin mono or aspirin mono (plain only, w/o FR) had slightly lower incidence rates for drug-related all GI AEs and minor GI AEs than placebo subjects.

In the aspirin FR group, the incidence rates of subjects with at least one drug-unrelated all GI AEs and minor GI AEs were slightly higher in subjects on aspirin FR than in those on regular aspirin or placebo. Drug-unrelated dyspepsia did not occur in any treatment group. Interestingly, in the aspirin FR group were absolutely no cases of drug-related GI based AEs (Table 5).

**Table 5:** Number of subjects (%) with at least one adverse event separated by treatment and dose group—sore throat*

	Aspirin mono			Aspirin + PSE			Aspirin mono, plain only, w/o FR			Aspirin FR			Placebo
	0–500 mg	501–1000 mg	Total	0–500 mg	501–1000 mg	Total	0–500 mg	501–1000 mg	Total	0–500 mg	501–1000 mg	Total	Total
Number of subjects treated	272	280	552	164	165	329	272	206	478	0	71	71	574
Number of subjects with at least 1 AE	99 (36.40)	15 (5.36)	114 (20.65)	8 (4.88)	17 (10.30)	25 (7.60)	99 (36.40)	1 (0.49)	100 (20.91)	0 (0.00)	13 (18.31)	13 (18.31)	96 (16.72)
Drug-unrelated													
All AEs	3 (1.10)	14 (5.00)	17 (3.08)	3 (1.83)	5 (3.03)	8 (2.43)	3 (1.10)	1 (0.49)	4 (0.84)	0 (0.00)	13 (18.31)	13 (18.31)	21 (3.66)
All GI AEs	0 (0.00)	4 (1.43)	4 (0.72)	2 (1.22)	2 (1.21)	4 (1.22)	0 (0.00)	1 (0.49)	1 (0.21)	0 (0.00)	3 (4.23)	3 (4.23)	5 (0.87)
Dyspepsia	0 (0.00)	0 (0.00)	0 (0.00)	0 (0.00)	0 (0.00)	0 (0.00)	0 (0.00)	0 (0.00)	0 (0.00)	0 (0.00)	0 (0.00)	0 (0.00)	0 (0.00)
Minor GI AEs	0 (0.00)	2 (0.71)	2 (0.36)	0 (0.00)	1 (0.61)	1 (0.30)	0 (0.00)	0 (0.00)	0 (0.00)	0 (0.00)	2 (2.82)	2 (2.82)	1 (0.17)
Drug-related													
All AEs	96 (35.26)	1 (0.36)	97 (17.57)	5 (3.05)	12 (7.27)	17 (5.17)	96 (35.29)	0 (0.00)	96 (20.08)	0 (0.00)	0 (0.00)	0 (0.00)	75 (13.07)
All GI AEs	3 (1.10)	1 (0.36)	4 (0.72)	2 (1.22)	4 (2.42)	6 (0.74)	3 (1.10)	0 (0.00)	3 (0.63)	0 (0.00)	0 (0.00)	0 (0.00)	13 (2.26)
Dyspepsia	3 (1.10)	1 (0.36)	4 (0.72)	0 (0.00)	0 (0.00)	0 (0.00)	3 (1.10)	0 (0.00)	3 (0.63)	0 (0.00)	0 (0.00)	0 (0.00)	4 (0.70)
Minor GI AEs	0 (0.00)	0 (0.00)	0 (0.00)	0 (0.00)	3 (1.82)	3 (0.91)	0 (0.00)	0 (0.00)	0 (0.00)	0 (0.00)	0 (0.00)	0 (0.00)	5 (0.87)

*Sore throat includes following indications: sore throat, sore throat and nasal congestion, nasal obstruction and pain (sore throat and/or headache), painful pharyngitis (sore throat pain)

In the dental pain model, the incidence rates of subjects with at least one drug-unrelated or –related GI based AEs were comparable after treatment with aspirin mono or placebo. Subjects on aspirin FR had also comparable incidence rates for drug-unrelated all GI AEs and minor GI AEs with those on aspirin mono or placebo, but had lowest incidence rates for drug-related GI based AEs. Drug-unrelated dyspepsia occurred only in very few cases and no subject suffered from drug-related dyspepsia (Table 6).

**Table 6:** Number (%) of adverse events separated by treatment and dose group—dental pain

	Aspirin mono			Aspirin + PSE			Aspirin mono, plain only, w/o FR			Aspirin FR			Placebo
	0–500 mg	501–1000 mg	Total	0–500 mg	501–1000 mg	Total	0–500 mg	501–1000 mg	Total	0–500 mg	501–1000 mg	Total	Total
Number of subjects treated	0	1065	1065	0	0	0	0	455	455	0	610	610	359
Number of subjects with at least 1 AE	0 (0.00)	156 (14.65)	156 (14.65)	0 (0.00)	0 (0.00)	0 (0.00)	0 (0.00)	71 (15.60)	71 (15.60)	0 (0.00)	85 (13.93)	85 (13.93)	70 (19.50)
Drug-unrelated													
All AEs	0 (0.00)	129 (12.11)	129 (12.11)	0 (0.00)	0 (0.00)	0 (0.00)	0 (0.00)	54 (11.87)	54 (11.87)	0 (0.00)	75 (12.30)	75 (12.30)	52 (14.48)
All GI AEs	0 (0.00)	43 (4.04)	43 (4.04)	0 (0.00)	0 (0.00)	0 (0.00)	0 (0.00)	12 (2.64)	12 (2.64)	0 (0.00)	31 (5.08)	31 (5.08)	25 (6.96)
Dyspepsia	0 (0.00)	1 (0.09)	1 (0.09)	0 (0.00)	0 (0.00)	0 (0.00)	0 (0.00)	0 (0.00)	0 (0.00)	0 (0.00)	1 (0.16)	1 (0.16)	1 (0.28)
Minor GI AEs	0 (0.00)	32 (3.00)	32 (3.00)	0 (0.00)	0 (0.00)	0 (0.00)	0 (0.00)	8 (1.76)	8 (1.76)	0 (0.00)	24 (3.93)	24 (3.93)	20 (5.57)
Drug-related													
All AEs	0 (0.00)	32 (3.00)	32 (3.00)	0 (0.00)	0 (0.00)	0 (0.00)	0 (0.00)	20 (4.40)	20 (4.40)	0 (0.00)	12 (1.97)	12 (1.97)	22 (6.13)
All GI AEs	0 (0.00)	14 (1.31)	14 (1.31)	0 (0.00)	0 (0.00)	0 (0.00)	0 (0.00)	8 (1.76)	8 (1.76)	0 (0.00)	6 (0.98)	6 (0.98)	7 (1.95)
Dyspepsia	0 (0.00)	0 (0.00)	0 (0.00)	0 (0.00)	0 (0.00)	0 (0.00)	0 (0.00)	0 (0.00)	0 (0.00)	0 (0.00)	0 (0.00)	0 (0.00)	0 (0.00)
Minor GI AEs	0 (0.00)	14 (1.31)	14 (1.31)	0 (0.00)	0 (0.00)	0 (0.00)	0 (0.00)	8 (1.76)	8 (1.76)	0 (0.00)	6 (0.98)	6 (0.98)	7 (1.95)

In the healthy volunteer model, subjects on aspirin mono and aspirin mono (plain only, w/o FR) might have more drug-unrelated and—related all AEs or GI based AEs in the higher dose than in the lower dose group. Drug- unrelated or—related GI based AEs hardly occurred in the aspirin + PSE and aspirin FR group. Drug-unrelated and—related dyspepsia as well as drug-related minor GI AEs were not found in aspirin FR. Interestingly, healthy subjects on placebo had the highest incidence rates for drug-related all AEs or GI based AEs (Table 7).

**Table 7:** Number (%) of adverse events separated by treatment and dose group—healthy volunteers

	Aspirin mono			Aspirin + PSE			Aspirin mono, plain only, w/o FR			Aspirin FR			Placebo
	0–500 mg	501–1000 mg	Total	0–500 mg	501–1000 mg	Total	0–500 mg	501–1000 mg	Total	0–500 mg	501–1000 mg	Total	Total
Number of subjects treated	557	502	1059	108	0	108	273	163	436	74	41	115	281
Number of subjects with at least 1 AE	44 (7.90)	105 (20.92)	149 (14.07)	16 (14.81)	0 (0.00)	16 (14.81)	16 (5.89)	36 (22.09)	52 (11.93)	11 (14.86)	1 (2.44)	12 (10.43)	90 (32.03)
Drug-unrelated													
All AEs	28 (5.03)	59 (11.75)	87 (8.22)	12 (11.11)	0 (0.00)	12 (11.11)	10 (3.66)	32 (19.63)	42 (9.63)	9 (12.16)	1 (2.44)	10 (8.70)	47 (16.73)
All GI AEs	3 (0.54)	21 (4.18)	24 (2.27)	1 (0.93)	0 (0.00)	1 (0.93)	1 (0.37)	9 (5.52)	10 (2.29)	2 (2.70)	0 (0.00)	2 (1.74)	10 (3.56)
Dyspepsia	0 (0.00)	8 (1.59)	8 (0.76)	0 (0.00)	0 (0.00)	0 (0.00)	0 (0.00)	0 (0.00)	0 (0.00)	0 (0.00)	0 (0.00)	0 (0.00)	3 (1.07)
Minor GI AEs	1 (0.18)	11 (2.19)	12 (1.13)	0 (0.00)	0 (0.00)	0 (0.00)	0 (0.00)	9 (5.52)	9 (2.06)	1 (1.35)	0 (0.00)	1 (0.87)	8 (2.85)
Drug-related													
All AEs	16 (2.87)	57 (11.35)	73 (6.89)	5 (4.63)	0 (0.00)	5 (4.63)	6 (2.20)	8 (4.91)	14 (3.21)	2 (2.70)	0 (0.00)	2 (1.74)	49 (17.44)
All GI AEs	5 (0.90)	50 (9.96)	55 (5.19)	2 (1.85)	0 (0.00)	2 (1.85)	3 (1.10)	6 (3.68)	9 (2.06)	1 (1.35)	0 (0.00)	1 (0.87)	45 (16.01)
Dyspepsia	0 (0.00)	28 (5.58)	28 (2.64)	0 (0.00)	0 (0.00)	0 (0.00)	0 (0.00)	0 (0.00)	0 (0.00)	0 (0.00)	0 (0.00)	0 (0.00)	29 (10.32)
Minor GI AEs	1 (0.18)	11 (2.19)	12 (1.13)	1 (0.93)	0 (0.00)	1 (0.93)	1 (0.37)	6 (3.68)	7 (1.61)	0 (0.00)	0 (0.00)	0 (0.00)	8 (2.85)

In both pain models and in the healthy volunteers group, subjects on aspirin FR had obviously less drug-related AEs and especially drug-related GI based AEs compared to placebo. Subjects on aspirin FR had no drug-related dyspepsia in any pain model and in the healthy volunteers group. However, significant differences in the occurrence of (GI) AEs between these treatment groups could not be evaluated in this descriptive model.

## Discussion

The most important findings from our study were that subjects treated with aspirin FR tablets had considerably less all AEs, drug-related all AEs, and all GI AEs compared to those on placebo. It might seem curious at first sight that (drug-related) AEs in the aspirin FR group occurred less frequently than in the placebo group. A reason for this might be the very fast pain relief induced by aspirin FR tablets leading to a subjectively better sense of well-being in general compared to subjects in pain who did not receive any pain killer and overall felt uncomfortable. Interestingly, healthy subjects also had more drug-related AEs in the placebo than in the aspirin FR group which also might be explained by the fast pain-relief in the aspirin FR group leading to a higher sense of well-being compared to healthy subjects without any symptoms who maybe are more focused on even small physical changes induced by the treatment.

Additionally, subjects on regular formulations of aspirin had a significantly increased risk of drug-related all AEs compared to placebo. However, we only found differences in the safety profile between subjects on aspirin and those on placebo, but not between subjects on regular formulations of aspirin and aspirin FR. Notably, drug-related dyspepsia and GI bleeding did not occur in any subject treated with aspirin FR tablets and we only found very few cases in the regular aspirin group.

Finally, the dose of regular aspirin and aspirin FR had no impact on the occurrence of drug—unrelated and—related (GI) AEs.

Our meta-analysis is an update of two previous small efficacy studies included about 400 subjects each suffering from dental pain. The safety profile was evaluated of two doses (study 1: 650 mg and study 2: 1000 mg) of regular formulations of aspirin and aspirin FR, respectively. They found that incidence rates of subjects with at least one AE or GI disorders were lower after intake of aspirin FR compared to placebo. Aspirin FR did not show a better safety profile compared to regular galenic formulations of aspirin. These results applied for both dose groups and the analyses were not performed for drug-related AEs (Cooper and Voelker 2012). These findings perfectly fit to our results from the risk analysis. In the descriptive analysis, subjects suffering from dental pain and taking aspirin FR had almost the same incidence rates for drug-unrelated all AEs or GI based AEs compared to placebo, but also show a comparable safety profile to subjects on regular aspirin. In addition, we also did not find any effect of the aspirin dose on the occurrence of AEs.

We complemented these findings by showing that subjects on aspirin FR had a lower risk of drug-related all AEs whereas subjects on regular formulations of aspirin had a higher risk of them compared to placebo. But again, subjects on aspirin FR and those on regular formulations of aspirin did not differ in the risk of drug-related events. In the dental pain model, subjects on aspirin FR had the lowest incidence rates for drug-related all AEs or GI based AEs.

### Pharmacokinetic and efficiacy of aspirin FR tablets compared to regular aspirin galenics and other analgesics

Although we found that subjects on aspirin FR tablets had a similar safety profile as those on regular galenic formulations of aspirin, aspirin FR has better pharmacokinetic characteristics compared to regular aspirin due to its galenic improvement. Regular galenic formulations of aspirin had a higher time to reach maximal concentration (*t*_max_) and a lower maximal concentration (*C*_max_) than aspirin FR which is characterized by a faster disintegraton and dissolution (Schick et al. 2020).

As expected, the improved galenic formulation of aspirin FR tablets showed a faster pain relief than regular galenic formulations. In two clinical trials including about 400 subjects each, subjects suffering from dental pain were treated with aspirin FR tablets, regular galenic formulations of aspirin or placebo (study 1: 650 mg and study 2: 1000 mg). Median time to first perceptible pain relief (FPR) and meaningful pain relief (MPR) were significiantly lower in subjects treated with 650 mg/1000 mg of aspirin FR tables compared to those treated with 650 mg/1000 mg of regular formulations of aspirin and compared to those who took placebo. These studies clearly demonstrated that the analgesic efficacy is strongly enhanced by the refined galenic formulation in aspirin FR tablets (Cooper and Voelker 2012).

In two small studies including subjects suffering from either acute dental pain (*N* = 510) or acute sore throat pain (*N* = 177) receiving a single dose of 1000 mg of either aspirin FR or paracetamol tablets, the median time to MPR and to FPR were not statistically different between aspirin FR and paracetamol, but both each were significantly different from placebo. (Voelker et al. 2016).

Pharmacokinetic advantages of aspirin FR compared to ibuprofen were shown in a study including 12 healthy male volunteers. This study investigated the in vivo disintegration behaviour of either aspirin FR or ibuprofen tablets using pharmacoscintigraphy. This study reported that the time to complete disintegration was four to eight times faster for 500 mg and 1000 mg aspirin FR, respectively, compared to 400 mg ibuprofen/400 mg ibuprofen-lysin. The fast dispersible and dissolving aspirin FR with fast bioavailability led to less active ingredients particles adherence to the muscosa which might cause improved gastric tolerability (Stevens et al. 2019).

However, our study did not reveal a better gastric tolerability of aspirin FR compared to regular formulations of aspirin after short-term use. There might be two reasons for this finding: (a) the improved galenic formulation of aspirin FR does not lead to a better GI tolerability, or (b) an improved GI tolerability of aspirin FR is only noticeable after long-term dosing which has to be investigated in further studies.

### Safety profile of aspirin compared to other analgesics

Data comparing the safety profile of aspirin FR tablets with other analgesics are currently very rare. In two small studies, patients received a single dose of 1000 mg aspirin FR, paracetamol, or placebo. The patients suffered either from postoperative dental pain (*N* = 510) or sore throat pain (*N* = 177). Both studies showed that the incidence rates of both subjects with at least one AE of any kind and of GI nature were comparable between the aspirin FR group and the paracetamol group, but were always lower than in the placebo group (Voelker et al. 2016). However, these studies did not differentiate between AEs and drug-related AEs, which might reveal a better tolerability of aspirin FR tablets compared to paracetamol. Therefore, further studies are necessary to evaluate the tolerability of aspirin FR in comparison with other analgesic after short- and long-term treatment.

Strengths and limitations of this study are given in Online Resource 5.

In conclusion, GI tolerability of aspirin FR tablets and regular galenic formulations of aspirin were comparable after short-term use. However, subjects on aspirin FR had a significantly better and subjects on regular formulations of aspirin a significantly worse safety profile than those on placebo. Major GI complication did not occur after any galenic formulation of aspirin.

## Data Availability

This study is a meta-analysis of individual data obtained from all studies conducted by Bayer between 2006 and 2020, a meta-analysis by Lanas et al. (2011) and 17 further studies on aspirin.
